# Ethanol exposure disrupts extraembryonic microtubule cytoskeleton and embryonic blastomere cell adhesion, producing epiboly and gastrulation defects

**DOI:** 10.1242/bio.20135546

**Published:** 2013-08-14

**Authors:** Swapnalee Sarmah, Pooja Muralidharan, Courtney L. Curtis, Jeanette N. McClintick, Bryce B. Buente, David J. Holdgrafer, Osato Ogbeifun, Opeyemi C. Olorungbounmi, Liliana Patino, Ryan Lucas, Sonya Gilbert, Evan S. Groninger, Julia Arciero, Howard J. Edenberg, James A. Marrs

**Affiliations:** 1 Department of Biology, Indiana University-Purdue University Indianapolis, 723 West Michigan Street, Indianapolis, IN 46202-5130, USA; 2 Department of Biochemistry and Molecular Biology, Indiana University School of Medicine, Indianapolis, IN 46202, USA; 3 Department of Mathematics, Wabash College, Crawfordsville, IN 47933, USA; 4 Department of Mathematics, Indiana University-Purdue University Indianapolis, Indianapolis, IN 46202, USA

**Keywords:** Fetal alcohol spectrum disorder, Gastrulation, Cell adhesion, Zebrafish

## Abstract

Fetal alcohol spectrum disorder (FASD) occurs when pregnant mothers consume alcohol, causing embryonic ethanol exposure and characteristic birth defects that include craniofacial, neural and cardiac defects. Gastrulation is a particularly sensitive developmental stage for teratogen exposure, and zebrafish is an outstanding model to study gastrulation and FASD. Epiboly (spreading blastomere cells over the yolk cell), prechordal plate migration and convergence/extension cell movements are sensitive to early ethanol exposure. Here, experiments are presented that characterize mechanisms of ethanol toxicity on epiboly and gastrulation. Epiboly mechanisms include blastomere radial intercalation cell movements and yolk cell microtubule cytoskeleton pulling the embryo to the vegetal pole. Both of these processes were disrupted by ethanol exposure. Ethanol effects on cell migration also indicated that cell adhesion was affected, which was confirmed by cell aggregation assays. E-cadherin cell adhesion molecule expression was not affected by ethanol exposure, but E-cadherin distribution, which controls epiboly and gastrulation, was changed. E-cadherin was redistributed into cytoplasmic aggregates in blastomeres and dramatically redistributed in the extraembryonic yolk cell. Gene expression microarray analysis was used to identify potential causative factors for early development defects, and expression of the cell adhesion molecule protocadherin-18a (*pcdh18a*), which controls epiboly, was significantly reduced in ethanol exposed embryos. Injecting *pcdh18a* synthetic mRNA in ethanol treated embryos partially rescued epiboly cell movements, including enveloping layer cell shape changes. Together, data show that epiboly and gastrulation defects induced by ethanol are multifactorial, and include yolk cell (extraembryonic tissue) microtubule cytoskeleton disruption and blastomere adhesion defects, in part caused by reduced *pcdh18a* expression.

## Introduction

Fetal alcohol spectrum disorder (FASD) is the most frequent preventable birth defect syndrome. Medical treatment, special education and lost productivity place a heavy toll on society (Popova et al., 2011). Developmental defects produced by ethanol exposure in animal models recapitulate defects seen in human FASD patients, including craniofacial, neural and cardiac defects (Ali et al., 2011; Haycock, 2009; Kelly et al., 2009; Sarmah and Marrs, 2013). Ethanol exposure could disrupt one or many developmental signaling mechanisms during early development (Muralidharan et al., 2013). Ethanol exposure is thought to affect embryonic gene expression, although mechanisms and specific changes are largely unknown (Haycock, 2009).

Gastrulation is a critical step in embryogenesis, producing the major embryonic axes (dorsal–ventral, anterior–posterior and left–right) (Solnica-Krezel and Sepich, 2012). Gastrulation is particularly sensitive to teratogen exposure (Gilbert-Barness, 2010), and ethanol exposure during gastrulation affects embryonic cell movements (Blader and Strähle, 1998; Sulik et al., 1981). Zebrafish (*Danio rerio*, Hamilton) is an exceptionally powerful model for studying gastrulation due to its rapid development, genetics, genomics, small size and experimental accessibility (Solnica-Krezel, 2006; Solnica-Krezel and Sepich, 2012). For example, injection of specific synthetic mRNA in early zebrafish embryos can rescue individual gene mutations or morpholino oligonucleotide gene knockdowns. Synthetic *shh* mRNA injection rescued effects of ethanol exposure in zebrafish (Loucks and Ahlgren, 2009).

Early embryogenesis includes cleavage and gastrulation stages. In zebrafish, epiboly cell movements occur during these stages, as blastomeres spread over the large yolk cell (Warga and Kimmel, 1990). Epiboly is coupled with gastrulation (Solnica-Krezel and Driever, 1994; Strähle and Jesuthasan, 1993). Previously characterized epiboly mechanisms include: (i) yolk cell microtubule cytoskeleton pulling of the outer layer of cells, the enveloping layer, towards the vegetal pole (Solnica-Krezel and Driever, 1994; Strähle and Jesuthasan, 1993); and (ii) radial intercalation, where blastomere (deep) cells move and intercalate radially in the spherical embryo, causing blastomere layer thinning (Kane et al., 2005; Morita and Heisenberg, 2013; Solnica-Krezel, 2006; Song et al., 2013). Classical experiments showed that epiboly activities in the yolk cell are independent of the deep cell movements since yolk cell epiboly processes proceeded when the embryonic blastomeres were removed from the yolk cell (Betchaku and Trinkaus, 1978).

E-cadherin adhesion activity is required for epiboly and convergence/extension cell movements during gastrulation (Babb and Marrs, 2004; Kane et al., 2005; Morita and Heisenberg, 2013; Solnica-Krezel, 2006; Song et al., 2013). E-cadherin distribution and trafficking is regulated during gastrulation, particularly in the prechordal plate (the leading edge of the anterior mesendoderm) by critical early developmental signaling pathways, including non-canonical Wnt (Ulrich et al., 2005), heterotrimeric G-protein (Lin et al., 2009) and Pou5f1/Oct4 signaling pathways (Song et al., 2013).

Cell labeling, marker expression, protein distribution and live embryo imaging experiments were performed to dissect effects of ethanol on epiboly and gastrulation. Microtubule cytoskeleton in the yolk cell was disrupted by ethanol exposure, indicating that extraembryonic tissue effects contribute to early ethanol-sensitive developmental defects. Blastomere cell directional movements and cell adhesion activity was reduced by ethanol exposure, but ethanol effects on E-cadherin expression and distribution in blastomeres were minimal. Microarray analysis showed reduced *pcdh18a* gene expression in ethanol treated embryos, and *pcdh18a* mRNA injection partially rescued epiboly defects, showing that reduced protocadherin cell adhesion molecule expression is partially responsible for the complex effects of ethanol on zebrafish early embryo development.

## Results

**Fig. 1.**
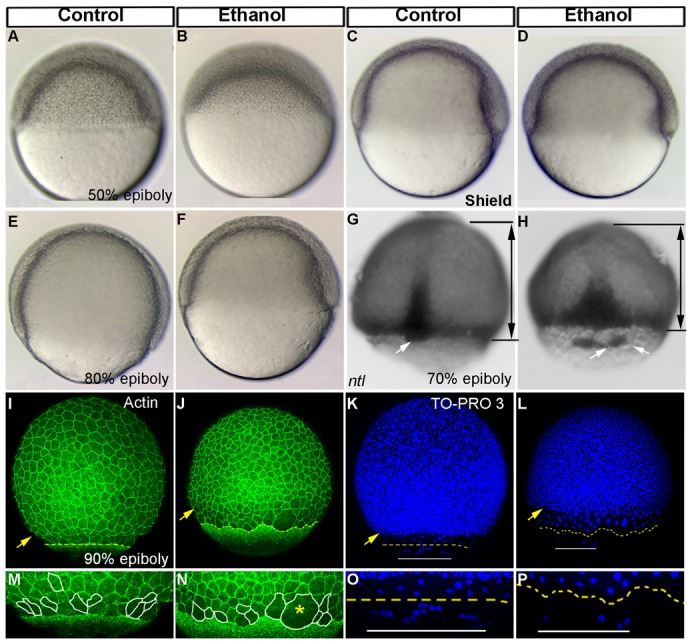
Ethanol exposure reduces epiboly progression and dorsal forerunner cell aggregation. (A–F) Live embryos at 50% epiboly (A,B), shield (C,D), and 80% epiboly stages (E,F) showed reduced epiboly progression in the ethanol treated embryos (B,D,F) compared to control (A,C,E). (G,H) *In situ* hybridization depicting *ntl* showed epiboly delay in the deep cells and obvious separation of the dorsal forerunner cells from the deep cell margin in the ethanol treated embryo (H). Black lines with arrows indicate the distance between the deep cell margin and the animal pole. White arrows: dorsal forerunner cells. (I,J) 3D renderings of confocal microscopy optical sections of phalloidin stained (F-actin) gastrulae. Yellow arrowhead: deep cell margin; yellow perforated line: EVL margin. (K,L) 3D renderings of confocal microscopy optical sections of TO-PRO-3 stained embryos showed deep cells nuclei, EVL cell nuclei and YSL nuclei. Yellow arrowhead: deep cell margin; yellow perforated line: EVL margin drawn from F-actin staining (I,J); white line: yolk syncytial nuclei margin. (M,N) High magnification images of control and ethanol treated embryos highlighting cell boundaries of a few EVL cells. Cells at the embryo margins in the control embryo showed elongated EVL cells, roughly perpendicularly aligned to the EVL margin (M). Ethanol treated embryos showed rounder and not correctly aligned EVL cells (N). Yellow asterisk indicates big multinucleated cell. (O,P) High magnification images of the TO-PRO-3 stained control and ethanol treated embryos. Control embryos showed YSL nuclei proceeded beyond the EVL (O). Ethanol treated embryos showed fewer YSL nuclei proceeded beyond the EVL.

Zebrafish embryos were treated with 100 mM ethanol in embryo medium beginning at 2 hpf; controls without ethanol were examined in parallel. This ethanol concentration produces highly reproducible phenotypes and is within levels attained in alcoholic patients. Epiboly progression was slowed (Fig. 1A–F; statistical comparison in Fig. 5F). Using *no tail (ntl) in situ* hybridization to mark the germ ring (70% epiboly stage; mesendodermal cells at the leading edge during epiboly progression) illustrates delayed epiboly progression (Fig. 1G,H). Dorsal forerunner cells (also marked by *ntl* staining) associate closely with the leading edge of the germ ring in control embryos, but these cells were dissociated from one another and advancing ahead of the germ ring in ethanol treated embryos at 6 hpf (Fig. 1G,H). Time lapse analysis of cell movements showed that the dorsal forerunner cells reaggregate by 10 hpf even in the continual presence of ethanol, and eventually form a single Kupffer's vesicle at 12 hpf (S.S., C.L.C. and J.A.M., unpublished observations). Primary cilia assembly in the Kupffer's vesicle, left–right asymmetry marker expression and heart looping were not disrupted in ethanol treated embryos (S.S. and J.A.M., unpublished observations; Sarmah and Marrs, 2013).

### Microtubule cytoskeleton distribution was disrupted in ethanol treated embryos

Epiboly can be controlled by purse string (actinomyosin) constriction and flow friction mechanisms, pulling the enveloping layer (which overlies and envelops the deep cell layer) toward the vegetal pole after 50% epiboly (Behrndt et al., 2012; Cheng et al., 2004; Köppen et al., 2006; Lin et al., 2009; Zalik et al., 1999). Actin and nuclear staining were used to mark the embryo location and evaluate cell shapes and cytoskeletal distribution. Actin cytoskeleton associated with the enveloping layer was present in ethanol treated embryos, similar to control embryos (90% epiboly stage; Fig. 1I,J,M,N). Yolk syncytial nuclei movements proceeded beyond the enveloping layer in control embryos, but these nuclei movements were reduced in ethanol treated embryos (Fig. 1K,L,O,P), suggesting that the epiboly movements of deep cells and the extraembryonic yolk cell nuclei were disrupted.

Tension created by epiboly movement produces long and thin cells in the enveloping layer at the embryo margin (Lin et al., 2009). Ethanol treated embryos had larger (sometimes multinucleate) enveloping layer cells at the embryo margins (90% epiboly stage; Fig. 1M,N) that had lower length-to-width ratios than control embryo cells (Fig. 5G); the ratio changed from 1.87 (s.d. = 0.69, *n* = 30) in control embryos to 1.00 (s.d. = 0.47, *n* = 33) in ethanol treated embryos (*P*-value <0.001), suggesting that there was less tension pulling the enveloping layer in ethanol treated embryos. Since the circumferential actin cytoskeleton was relatively normal in ethanol treated embryos, the microtubule cytoskeleton in the yolk cell, which pulls the enveloping layer toward the vegetal pole, was evaluated next.

**Fig. 2.**
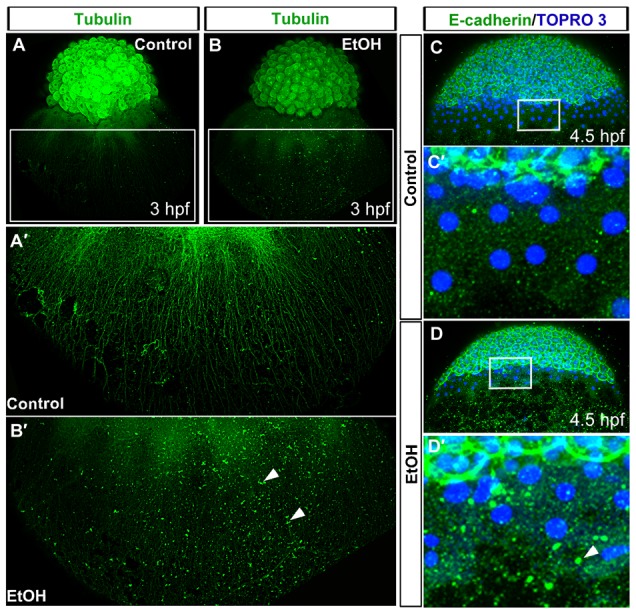
Ethanol exposure disrupts yolk cell microtubule and E-cadherin distribution. (A,B) 3D renderings of confocal microscopy optical sections of anti-α-tubulin antibody stained embryos showed microtubule organizations in the control (A) and ethanol treated (B) embryos at 3 hpf. (A′,B′) High magnification image of the boxed regions of embryos showed arrays of yolk cell microtubule extending toward the vegetal pole in the control (A′) and abnormal arrays of yolk cell microtubules in the ethanol treated embryo (B′). Arrowheads indicate aggregates of α-tubulin in the yolk cell. (C–D′) 3D images of confocal microscopy optical sections of anti-E-cadherin antibody stained embryos showed E-cadherin distribution in the control (C) and ethanol treated (D) embryos at 4.5 hpf. High magnification image of the boxed region of the control embryo showed relatively small aggregates of E-cadherin in the yolk cell (C′) than ethanol treated embryos, which had large aggregates of E-cadherin in the yolk cell (D′). Arrowhead indicates large E-cadherin aggregate.

Microtubule networks are associated with the enveloping layer, extending toward the vegetal pole of the yolk cell in 3 hpf embryos (Fig. 2A,B,A′,B′). These microtubules associate with the circumferential actin cytoskeleton at the enveloping layer margin. Disrupting these microtubules interferes with epiboly cell movements (Solnica-Krezel and Driever, 1994; Strähle and Jesuthasan, 1993). In ethanol treated embryos, the microtubule cytoskeleton displayed an abnormal distribution, having shorter microtubules, extending less distance toward vegetal regions of the yolk cell, indicating that ethanol disrupts microtubule arrays that drive epiboly cell movements.

The microtubule cytoskeleton in the yolk cell associates with E-cadherin, and loss of E-cadherin or microtubules disrupts this yolk cell membrane–cytoskeletal complex and interferes with epiboly cell movements (Babb and Marrs, 2004;  Carvalho et al., 2009; Kane et al., 2005; Solnica-Krezel and Driever, 1994). In ethanol treated embryos (4.5 hpf), there were large aggregates of E-cadherin staining in the yolk cell near the enveloping layer cell border (Fig. 2C,D,C′,D′) in contrast to E-cadherin distribution in the enveloping and deep cells of the embryo proper.

### Epiboly and gastrulation cell movement defects in ethanol treated embryos

**Fig. 3.**
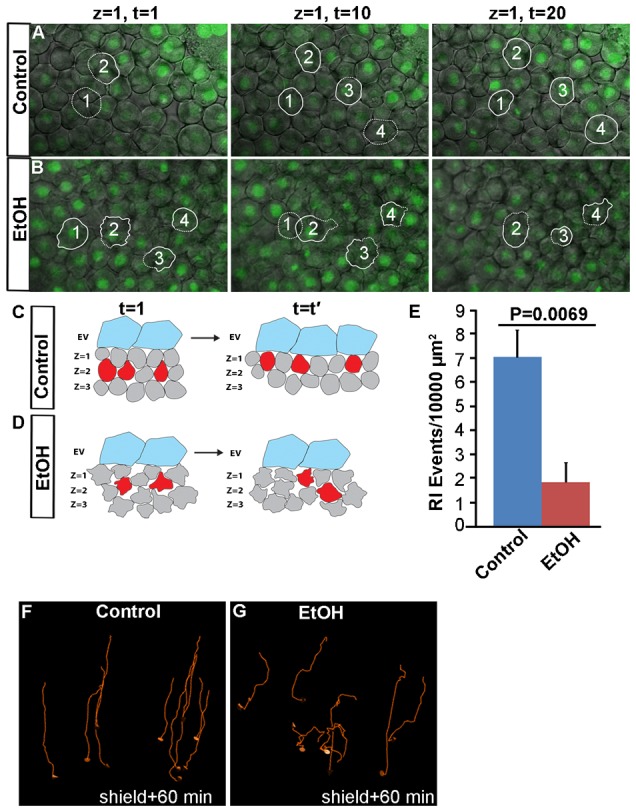
Ethanol exposure affects radial intercalation and gastrulation cell movements. (A,B) Confocal microscopy optical sections from the time-lapse image sequences at the most external epiblast layer of FITC-labeled histone-1 injected control (A) and ethanol (B) treated embryos at 4.5 hpf. Solid white line highlights representative cells in the upper layer, dotted line indicates cell in the lower layer. (C,D) Schematic diagrams illustrating radial intercalation (RI) events in the control (C) and ethanol treated (D) embryos. Unlike control, ethanol treated cells showed lamellipodia extension in all directions. (E) Histogram showing RI events in the control and ethanol treated embryos. (F,G) The paths of ten deep cells in the mesendoderm of control and ethanol treated embryos. Cells in ethanol treated embryos showed abnormal trajectories. Animal pole, top; vegetal pole, bottom.

A second mechanism that drives epiboly is radial intercalation cell movements, a process where cells at medial positions within the deep cell layer migrate radially and intercalate between cells within the outer and inner cell layers, producing a thinning and spreading of deep cells over the yolk cell (Morita and Heisenberg, 2013; Solnica-Krezel, 2006; Warga and Kimmel, 1990). Untreated control embryos showed more frequent radial intercalation events than ethanol treated embryos (Fig. 3; see also supplementary material Movies 1, 2). Migrating cells in control embryos directionally extended lamellipodia, leading to successful radial intercalation, but ethanol treated embryo cells extended lamellipodia in all directions and frequently failed to radially intercalate.

Cell movements in the shield, where mesendodermal cell involution first occurs during gastrulation, were examined from 6 to 7 hpf. Individual cell movements in control and ethanol treated embryos were directed toward the shield, and mesendodermal cells underwent involution (Fig. 3F,G). Deep cells in control and ethanol treated embryos moved at the same rate, having nearly identical average instantaneous velocities (Table 1). However, cells in ethanol treated embryos had abnormal paths, showing less directed movements toward the shield (Fig. 3F,G). These more random cell movements during gastrulation are reflected in a lower meandering index (Table 1; meandering index of 1.0 is a perfect straight line; the smaller the value, the greater the meandering of the track). The average meandering index was reduced to 0.770 in ethanol treated embryos from 0.959 in control embryos.

**Table 1.**
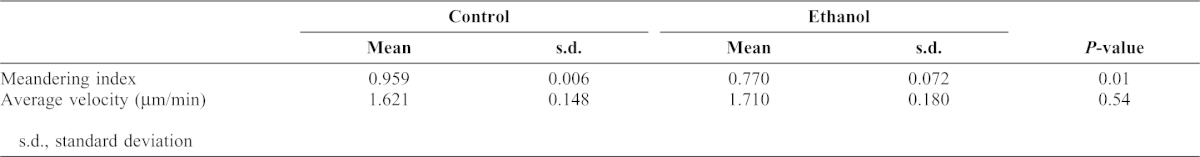
Ethanol interferes with directional gastrulation cell movements, but not migration rate.

### Adhesion was disrupted in ethanol treated embryos

**Fig. 4.**
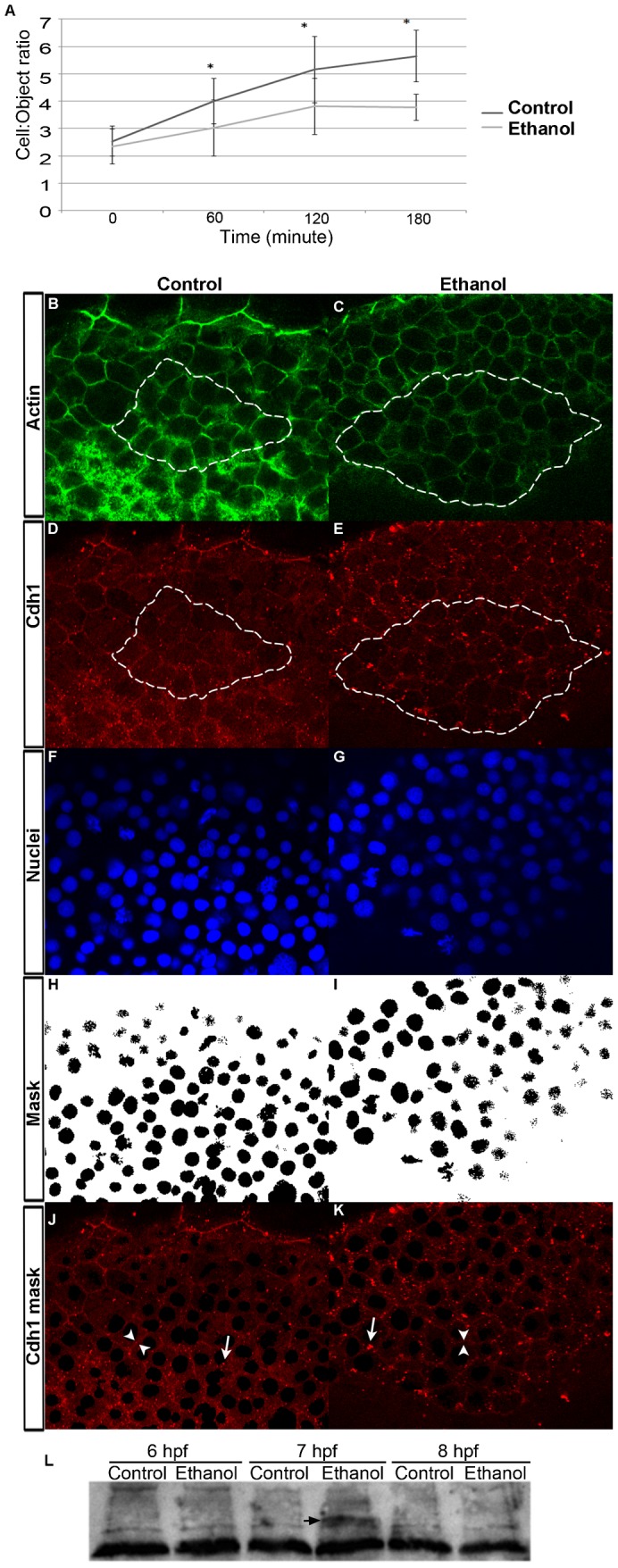
Ethanol treatment reduces blastomere adhesion, but E-cadherin expression and distribution were only minimally affected. (A) Cell adhesion assay using dissociated cells blastomeres. Graph showed cell-to-cell aggregation (object-to-cell ratios) of the dissociated control and ethanol treated blastomeres, which was reduced in ethanol treated cells. (B,C) Single confocal microscopy optical sections of the phalloidin stained prechordal plate cells in control and ethanol treated embryos at 8 hpf. (D,E) Single confocal microscopy optical sections showed E-cadherin distribution in the prechordal plate cells in the control and ethanol treated embryos at 8 hpf. White dotted lines indicate prechordal plate cells that were analyzed. (F,G) Co-staining with TO-PRO-3 labeled nuclei in the prechordal plate cells of control and ethanol treated embryos. (H,I) TO-PRO-3 was used as mask to exclude nuclei from E-cadherin intensity measurements. (J,K) E-cadherin distribution in the cytoplasm and cell surface after mask was applied. Arrowheads, cell surface; arrows, cytoplasmic aggregates. (L) Ethanol treatment did not change E-cadherin expression levels. Immunoblot analysis showed E-cadherin expression in the control and ethanol treated embryos at 6, 7 and 8 hpf. Arrow indicates an E-cadherin isoform that accumulated, which migrated more slowly.

To directly measure adhesion, dissociated cell aggregation assays using blastomeres from 4.5 hpf embryos were used to compare ethanol treated and control embryos. Cell aggregation was measured over a 3 hour time course and cell-to-object ratio was calculated. A ratio of 1.0 indicates no aggregation; greater the value, greater is the aggregation. Initially, the cell-to-object ratio was the same in control and ethanol treated cells (statistical comparison showed no significant difference at 1 hour), but at 2 and 3 hours, the cell-to-object ratio in control cells was significantly higher than ethanol treated cells (Fig. 4A).

Immunofluorescence using an E-cadherin specific antibody was performed on 8 hpf embryos that were treated with ethanol or untreated (Fig. 4D,E), and compared to cell surface and nuclei markers (Fig. 4B–K) in prechordal plate cells. E-cadherin distribution in the cytoplasm versus cell surface was not changed (Table 2), but cytoplasmic E-cadherin was found in larger cytoplasmic structures (see arrows in Fig. 4J,K). No difference in overall E-cadherin expression between control and ethanol treated embryos was detected at 6, 7 and 8 hpf (Fig. 4L). At 7 hpf, a band was consistently detected by the antibody that migrated more slowly in ethanol treated embryos. This may represent an accumulated E-cadherin isoform in ethanol exposed embryos (see arrow, Fig. 4L).

**Table 2.**
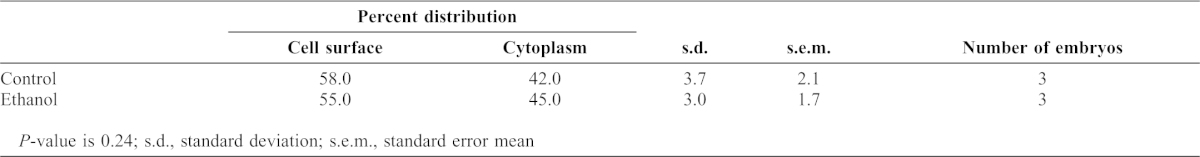
Cdh1 distribution at the cell surface versus cytoplasm was unaffected after ethanol exposure in 8 hpf embryos.

### Gene expression changes during gastrulation due to ethanol exposure

Gene expression changes were examined using Affymetrix GeneChip® microarray analysis. Many genes were identified that showed statistically significant changes in response to embryonic ethanol exposure between 2 and 8 hpf; a subset of those showing absolute changes ≥1.5-fold and P-values ≤0.001 are shown in Table 3 (the complete list is in supplementary material Table S1). Numerous genes were categorized by biological function based on published studies (see Materials and Methods), including cell adhesion (Table 3). Several gene expression changes were validated using quantitative PCR, indicating that the microarray analysis was sensitive and accurate (Table 4). There are functional categories, such as retinoid metabolism genes, that were expected based on previous studies. However, the dataset indicates that the transcriptional response to ethanol exposure in the early embryo is multifactorial.

**Table 3.**
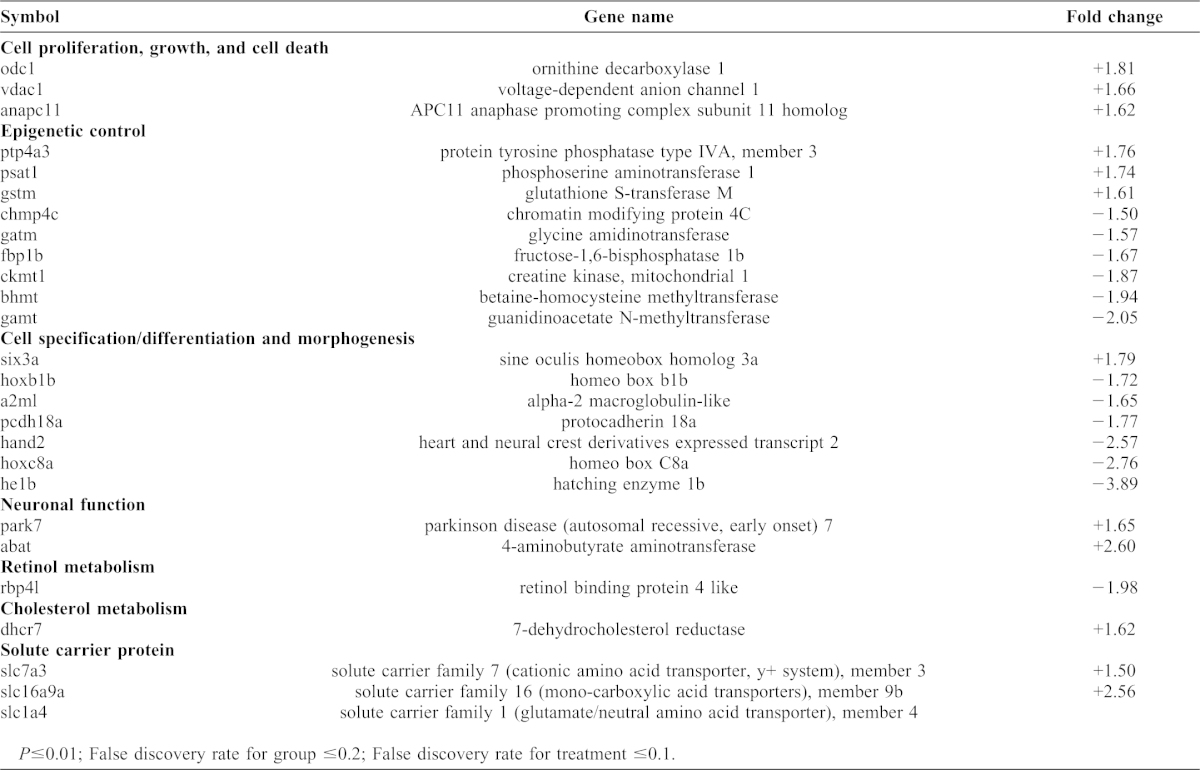
Ethanol responsive genes at 8 hpf. Expression level changes with fold change between ≤−1.50 or ≥1.5.

**Table 4.**
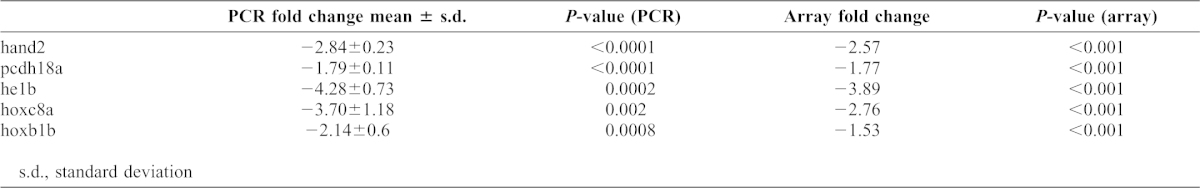
Quantitative PCR confirms decrease in expression of selected cell-specification/differentiation and morphogenesis genes at 8 hpf after ethanol treatment.

**Fig. 5.**
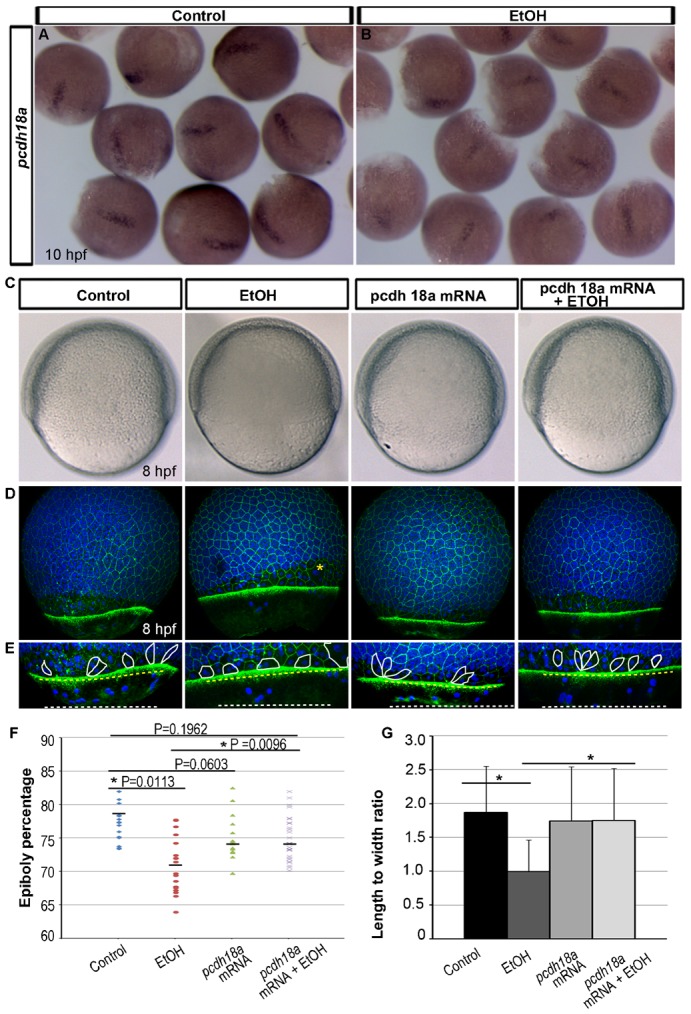
Ethanol induced epiboly defect was rescued by *pcdh18a* mRNA injection. (A,B) Whole mount *in situ* hybridization detecting *pcdh18a* mRNA showed reduced expression in the ethanol treated 10 hpf embryos as compared to control. (C) Bright field images focused at the EVL margin showed epiboly progression in the control, ethanol treated, *pcdh18a* mRNA injected, and *pcdh18a* mRNA injected plus ethanol treated embryos. (D) 3D renderings of confocal microscopy optical sections of phalloidin stained embryos co-labelled with TO-PRO-3 showed reduced epiboly in the ethanol treated embryos. Epiboly progression was similar in the control, *pcdh18a* mRNA injected, and *pcdh18a* mRNA injected plus ethanol treated embryos. Yellow asterisk indicates big multiucleated cells. (E) High magnification images of the embryos highlighting cell boundaries of a few EVL cells. Control, mRNA injected, and mRNA injected plus ethanol treated embryos showed elongated EVL cells; ethanol treated embryos showed rounder EVL cells that were not correctly aligned. Yellow perforated line: EVL margin; white line: yolk syncytial nuclei margin. Note: mRNA injected plus ethanol treated embryos showed more YSL nuclei beyond the EVL as compared to ethanol treated embryo. (F) Scatter plot representation shows reduced epiboly movement in ethanol treated embryos compared to control. These reduced epiboly movements were rescued by *pcdh18a* mRNA injection. (G) Histogram shows rescue by *pcdh18a* mRNA injection of the EVL cells length-to-width ratios at the embryo margins, which are reduced after ethanol treatment. Asterisks indicate statistical significance: *P<0.0001.

The gene encoding the adhesion molecules protocadherin-18a (*pcdh18a*) was identified, which showed reduced expression (1.77 fold by microarray analysis, and 1.79 fold by quantitative PCR analysis, Tables 3, 4). Previous studies showed that *pcdh18a* controls epiboly cell movements (Aamar and Dawid, 2008). Whole mount *in situ* hybridization showed that *pcdh18a* expression was generally reduced at all expression locations in ethanol treated 8 hpf embryos (Fig. 5A,B).

To test whether reduced *pcdh18a* expression caused by ethanol exposure produces epiboly delay in zebrafish embryos, synthetic *pcdh18a* mRNA was injected into 2-to-4 cell stage embryos, and these embryos were compared with uninjected embryos treated with ethanol or untreated. Epiboly progression was evaluated (Fig. 5C,F). Epiboly delay induced by ethanol was restored to near control levels by injection of synthetic *pcdh18a* mRNA (EtOH+mRNA) (Fig. 5C,F). Injecting the same concentration of *pcdh18a* mRNA in the absence of ethanol did not change epiboly progression (Fig. 5C,F). In addition, synthetic *pcdh18a* mRNA injection rescued enveloping layer cell shape changes in ethanol treated embryos, showing greater length-to-width ratios as compared with ethanol treatment alone (Fig. 5D,E,G). Yolk syncytial nuclei movements were partially rescued in the *pcdh18a* mRNA injected, ethanol treated embryos.

## Discussion

Birth defects are induced by various environmental factors. Despite *in utero* ethanol exposure being the most frequent preventable birth defect, mechanisms of ethanol toxicity remain poorly understood. Better understanding of the genesis of birth defect syndromes can help inform clinical therapeutic interventions. Zebrafish is emerging a useful model to examine environmental toxins and birth defect syndromes (Ali et al., 2011). Ethanol exposure produces a diverse spectrum of birth defects, which can be recapitulated using zebrafish, including craniofacial, neural and cardiac defects, which can have their origin during gastrulation stages (Dlugos and Rabin, 2010; Muralidharan et al., 2013; Sarmah and Marrs, 2013). Gastrulation stages are particularly critical for animal development. In vertebrates, gastrulation developmental events establish the body axes (Solnica-Krezel and Sepich, 2012). Ethanol was previously shown to disrupt gastrulation cell movements (Blader and Strähle, 1998; Yelin et al., 2005; Zhang et al., 2010). Our experiments establish that abnormal epiboly and gastrulation cell movements are associated with yolk cell microtubule cytoskeleton, radial intercalation cell movement, shield blastomere cell movement, blastomere cell adhesion and, specifically, *pcdh18a* expression defects. Partial, but significant rescue in *pcdh18a* mRNA injection experiments indicate that *pcdh18a* expression defect is part of the mechanism of ethanol teratogenesis, but there are likely to be many other defects that contribute to the overall ethanol exposure phenotype. In zebrafish, ethanol treatment disrupts the E-cadherin-microtubule network in the yolk cell, which appears also to contribute to teratogenesis.

### Adhesion defects in gastrulation

E-cadherin cell adhesion and other cell adhesion mechanisms control epiboly and radial intercalation cell movements in the deep cells or blastomeres (Aamar and Dawid, 2008; Hammerschmidt and Wedlich, 2008; Morita and Heisenberg, 2013; Solnica-Krezel, 2006; Ulrich et al., 2005). Gastrulation cell movements are also controlled by cell polarity mechanisms that respond to embryonic morphogen gradients, which often regulate differential cell adhesion in the early embryo (Roszko et al., 2009; Tada and Kai, 2012). Abnormal cell movements in the shield like those seen in ethanol treated embryos were also observed in embryos with cell adhesion defects, like E-cadherin loss-of-function embryos (Babb and Marrs, 2004; Kane et al., 2005). Our direct measurement in adhesion assays showed that ethanol affects cell adhesion activity. E-cadherin cell adhesion molecule expression and distribution in the cell surface versus cytoplasm was largely unaffected by ethanol exposure during early development. However, E-cadherin in the prechordal plate cells showed an abnormal distribution in larger cytoplasmic vesicles, and E-cadherin in the yolk cell was dramatically redistributed from the yolk cell surface associated with the embryo proper to large cytoplasmic vesicles. This redistribution coincides with microtubule redistribution in the yolk cell, and together, these effects could alter epiboly and gastrulation cell movements. Ethanol effects on *pcdh18a* gene expression may contribute to these cell adhesion and cell movement defects, perhaps playing an important role in ethanol induced zebrafish teratogenesis.

In zebrafish, E-cadherin adhesion during epiboly and gastrulation controls blastomere cohesion, and E-cadherin loss-of-function produces convergence/extension defects (Babb and Marrs, 2004; Kane et al., 2005). Mutations that cause epiboly defects were also identified in large scale screening, and *half-baked* mutations were found to be alleles of the E-cadherin gene, *cdh1* (Kane et al., 2005). Other gastrulation regulators, including Snail, Wnt11, heterotrimeric G-protein and Pou5f1 (Oct-4) signaling, regulate E-cadherin expression or intracellular trafficking (Ulrich et al., 2005; Esguerra et al., 2007; Lin et al., 2009; Speirs et al., 2010; Song et al., 2013).

Earlier studies showed that gastrulation stages are sensitive to ethanol exposure, producing persistent defects (Blader and Strähle, 1998; Zhang et al., 2010). Ethanol exposure during early development affects convergence/extension cell movements and prechordal plate migration. In zebrafish, these processes are coupled with epiboly movements. Our experiments confirm and support these previous findings (Blader and Strähle, 1998; Zhang et al., 2010), showing specific radial intercalation and shield cell migration defects. These cell migration defects were potentially caused by defects in cell adhesion mechanisms. Dissociated blastomere aggregation assays show that ethanol exposure reduced cell adhesion. Surprisingly, E-cadherin expression and distribution was largely unchanged. During early gastrulation, E-cadherin is needed to form the Kupffer's vesicle and establish left–right asymmetry in the embryo (Essner et al., 2005;  Kai et al., 2008; Matsui et al., 2011; Tay et al., 2013). Normal left–right asymmetry in ethanol exposed embryos (S.S. and J.A.M., unpublished observations; Sarmah and Marrs, 2013) supports our conclusion that there was not an overall E-cadherin deficiency.

### Ethanol induced gene expression changes: *pcdh18a*

To identify potential causative factors for the apparent adhesion defect in ethanol exposed embryos, gene microarray analysis was used to examine ethanol induced gene expression changes during gastrulation (8 hpf; mid-gastrulation). Hundreds of genes showed statistically significant expression changes, representing several functional categories including cell specification, differentiation, morphogenesis, proliferation, epigenetics, and retinol metabolism (Table 3). One category of genes that showed strong ethanol induced gene expression changes was solute transporter proteins (*slc* genes). It is interesting that *slc3a2* was shown to control yolk syncytial layer formation and microtubule distributions (Takesono et al., 2012). Analyzing the effects of solute transporter functional changes in response to ethanol exposure could be very instructive, particularly their effects on extraembryonic tissue development.

Previously, *shh* expression was shown to be reduced in ethanol treated zebrafish embryos (Loucks and Ahlgren, 2009), but our 8 hpf gene microarray did not detect differences in *shh* expression following ethanol treatment. A higher ethanol concentration (1–2.5%) was used in the previous study (Loucks and Ahlgren, 2009), as compared to 100 mM or 0.6% in our experiments.

Our study focused on cell adhesion functions. The cell adhesion molecule gene encoding Protocadherin-18a (*pcdh18a*) had reduced expression (Tables 3, 4). A previous study showed that Protocadherin-18a mediated adhesion regulates epiboly cell movements in the early embryo (Aamar and Dawid, 2008). The ability of synthetic *pcdh18a* mRNA injection experiments to partially rescue epiboly defects caused by ethanol illustrated that this gene plays a direct, mechanistic role in early development defects in the zebrafish FASD model. Protocadherin adhesion mechanisms are poorly understood. However, recent studies from Jontes and co-workers showed that protocadherin and cadherin proteins can physically interact, and the protocadherin/cadherin complex produces stronger adhesion than either adhesion molecule alone (Emond et al., 2011). Coordinated patterns of cell–cell adhesion control various cell movements during embryogenesis, including in the early gastrulation stage zebrafish embryo.

## Conclusions

Our analysis of ethanol effects on early development showed that yolk cell E-cadherin/microtubule cytoskeleton was disrupted; blastomere radial intercalation was reduced; and shield cell migration was disorganized. Defects in cell adhesion induced by ethanol partially explain these epiboly and gastrulation defects. Synthetic *pcdh18a* mRNA injection rescues ethanol induced epiboly defects, showing that *pcdh18a* expression changes participate in the pathogenesis in this zebrafish model of FASD. Ethanol effects on yolk cell microtubule cytoskeleton and E-cadherin distribution suggests that extraembryonic tissues in the early embryo also contribute to the FASD phenotype.

In addition to *pcdh18a*, numerous other genes were identified whose expression changed when embryos were exposed to ethanol during early development (Table 3). The data do not point to a single effect that can explain a majority of these gene changes. Ethanol responsive genes fall into various pathways and functional categories, including retinoid metabolism, solute transport and neurogenic functions. Some genes identified in our gene microarray experiments (Table 3) were functionally characterized in previous studies (Aamar and Dawid, 2008; Inbal et al., 2007; Lagutin et al., 2003). The central importance of pluripotency regulators, Notch, Wnt and other pathways represented in the microarray experiments strongly indicate that the approach produced important data to test future hypotheses.

## Materials and Methods

### Zebrafish husbandry and ethanol treatment

Zebrafish (*Danio rerio*; Hamilton; TL strain) were raised and housed under standard laboratory conditions (Westerfield, 2000) in accordance with Indiana University Policy on Animal Care and Use. Embryos were exposed to ethanol by incubation in embryo medium containing 100 mM (0.6% vol./vol.) ethanol from 2 hours post fertilization (hpf) until the completion of the experiment, in Petri dishes wrapped with parafilm and maintained at 28.5°C.

### Microscopy

Images of live embryos were collected using a Leica MZ12 microscope equipped with Leica DFC290 camera (Leica Microsystems Inc., Buffalo Grove, IL, USA). Confocal images were acquired using a Zeiss Observer Z1 LSM 700 confocal microscope (40× 1.1 NA W or 20× 0.8 NA objectives; Carl Zeiss Microscopy, Thornwood, NY, USA).

### Cell labeling and time-lapse imaging

Embryos (1–4 cell stages) were injected with FITC-conjugated histone-1 protein. Embryos were dechorionated and mounted in low melting agarose at 4.3 hpf or 6 hpf. Images at several focal planes were captured every 2 minutes for 1–3 hours using Zeiss Observer Z1 LSM 700 confocal microscope (40× 1.1 NA W); images were focused at the shield region in 6 hpf embryos. All time-lapse movies were processed using Volocity software (Perkin Elmer, Waltham, MA, USA). Radial intercalation cell movements were evaluated manually. Average instantaneous velocities and meandering indexes (relative displacement) were calculated using Volocity software.

### *In situ* hybridization

Whole-mount *in situ* hybridization of zebrafish embryos was performed as described (Sarmah et al., 2010). Digoxigenin-labeled riboprobes for *ntl* and *pcdh18a* (generously provided by Drs C. Nusslein-Volhard and I. Dawid, respectively) were synthesized using DIG RNA Labeling Kit (Roche, Indianapolis, IN, USA) according to manufacturer's recommendations. Images were collected using a Leica MZ12 microscope equipped with Leica DFC290 camera.

### Immunofluorescence, F-actin staining and image analysis

Whole-mount immunostaining was performed as previously described (Clendenon et al., 2012) using primary antibodies against E-cadherin (Cdh1) and α-tubulin, at a dilution of 1:500 in blocking solution. Texas red or Alexa 488-conjugated anti-rabbit and Alexa 488-conjugated anti-mouse secondary antibodies were used at a 1:100 dilution (Molecular probes/Invitrogen). Alexa 488-conjugated phalloidin (Molecular Probes/Life Technologies, Inc., Grand Island, NY, USA) was used at a 1:100 dilution. Nuclei were stained with TO-PRO-3 at a dilution of 1:1000.

Image analysis to measure cytoplasmic versus cell surface E-cadherin staining was performed as follows using Image J software. Embryos (8 hpf) were stained with E-cadherin antibody, phalloidin and TO-PRO-3 (Molecular Probes/Life Technologies, Inc.), and the prechordal plate region was imaged using confocal microscopy. Images containing the prechordal plate were identified in ethanol treated and control embryos. TO-PRO-3 images were used to make a mask that removed the nucleus area and staining from the E-cadherin. Actin staining was used to highlight the cell surface and cytoplasmic regions of the cells within the prechordal plate region. These highlighted regions were transferred to the corresponding E-cadherin image, and fluorescence intensity per unit area was calculated. Percentage of total staining at the cell surface versus cytoplasm were calculated in three independent experiments. Image J software was also used to measure length-to-width ratio of enveloping layer cells at the embryo margin.

### Cell adhesion assay

Embryos at 4.5 hpf were dechorionated and incubated in 0.05% trypsin–EDTA for 10 minutes. Cells were disassociated using a glass Pasteur pipette, passing the cell suspension through the pipette 15–25 times to produce a primarily single cell suspension. Rinsing with 10% fetal bovine serum (in L-15 medium) was used to stop the trypsin reaction. Cells were harvested by centrifugation (1,800 rpm) for 3 minutes at 22°C. Cells were resuspended in L-15 medium with or without ethanol, and plated on 10 µg/ml fibronectin-coated, chambered coverslip slides. Images were collected at the same locations over a 3-hour time course using Zeiss Observer Z1 (20× 0.8 NA objective) equipped with a robotic stage and an Orca-AG CCD camera (Hamamatsu Photonics, K. K., Bridgewater, NJ, USA). Cells and cell aggregates (objects) were counted, and a cell-to-object ratio was calculated at each time point.

### Immunoblotting

Immunoblotting was performed as previously described (Babb and Marrs, 2004). Cdh1 primary antibody was diluted 1:15,000, and anti-rabbit horseradish peroxidase (HRP)-conjugated secondary antibody (1:10,000 dilution; Amersham, Arlington Heights, IL, USA). Chemiluminescence (ECL kit, Amersham) according to manufacturer directions was used, and membranes were exposed to film (Kodak Bio-Max ML, Eastman Kodak, Rochester, NY, USA). Three independent experiments were compared.

### RNA isolation and quantitative PCR analysis

Total RNA was extracted from approximately 20 treated and untreated embryos at 8 hpf using TRIzol reagent (Sigma, St Louis, MO, USA). For quantitative PCR, 1 µg of total RNA was reverse transcribed to cDNA using M-MLV reverse transcriptase (Promega, Madison, WI, USA), and cDNA was diluted tenfold with RNase free water. Each 20 µl PCR reaction was performed with 1–4 µl of cDNA using Power SYBR Green PCR mix (Applied Biosystems/Life Technologies, Inc.) and 0.5 µM of each primer. Primer sets used are listed in supplementary material Table S2. Three independent experiments in triplicate were performed using *rsp15* as internal control with either the 7300 Real Time PCR System (Applied Biosystems) or the LightCycler 480 (Roche).

### Microarray analysis

Embryos were exposed to ethanol from 2 to 8 hpf or left untreated in seven independent experiments. Total RNA was extracted from approximately 20 treated and untreated embryos for each treatment in each experiment, using TRIzol reagent (Sigma). The RNA samples were examined for quality using the Agilent Bioanalyzer RNA Nanochip (Agilent Technologies, Santa Clara, CA, USA). The RIN (RNA integrity number) for all samples was ≥9.0. The samples were labeled using the standard protocol for the Affymetrix 3′IVT Express kit (Affymetrix, Santa Clara, CA, USA) starting with 100 ng of total RNA. Individual labeled samples were hybridized to the Zebrafish Genome Array (Affymetrix) for 17 hours then washed, stained and scanned following the standard protocol. All 14 arrays were labeled, hybridized and scanned in one batch. Arrays were visually scanned for abnormalities or defects; none were found.

Affymetrix gene expression console software was used to generate MAS5 (MicroArray Suite 5.0) signals and detection calls; arrays were scaled to a target of 1000. To avoid analyzing genes that were not expressed in any condition, only those probe sets that had a fraction present ≥0.40 in at least one of the two treatments were analyzed (McClintick and Edenberg, 2006). MAS5 signals were imported into Partek Genomics Suite (Partek, Inc., St Louis, MO, USA) and log_2_ transformed. These log_2_ transformed signals were used for Principal Components Analysis (PCA), hierarchical clustering and signal histograms to determine if there were any outlier arrays. No outliers were detected. The PCA plot and hierarchical clustering indicated that there was a batch effect associated with the batch of embryos (perhaps because each experiment was performed using embryos derived from a single breeding pair). The log_2_ transformed signals were analyzed using a 2-way ANOVA with factors for treatment (alcohol vs control) and embryo batch (random effect). This analysis indicated that the embryo batch was indeed significant. The False Discovery Rate (FDR) was calculated using the Storey qvalue method (Storey and Tibshirani, 2003). Microarray data were deposited in the NCBI GEO database, accession number GSE48380. Performing PubMed searches identified protein functions using the gene names with absolute changes ≥1.5-fold and P-values ≤0.001. Functional categories shared by 2 or more genes were listed in Table 3.

### mRNA injection

For *pcdh18a* mRNA rescuing experiments, mRNA was synthesized from a pCS2+ *pcdh18a* vector (Aamar and Dawid, 2008) using a SP6 mMessage mMachine kit (Ambion, Austin, TX, USA). Synthetic mRNA (75 pg/embryo) was injected into the embryos at 1-cell stage. Injected and uninjected embryos were treated with and without 100 mM ethanol from 2 to 8 hpf. These embryos were fixed, dechorionated and imaged focusing on enveloping cell layer at the embryo margin. Percent epiboly progression was calculated using Image J software.

### Statistical analysis

In addition to microarray statistical analyses, analyses on cell migration, cell adhesion, cell shape, protein distribution, qPCR and epiboly were performed using unpaired two-tailed Student's *t*-test (GraphPad Software, La Jolla, CA, USA).

## Supplementary Material

Supplementary Material
