# Supplementary material for: Ethanol exposure disrupts extraembryonic microtubule cytoskeleton and embryonic blastomere cell adhesion, producing epiboly and gastrulation defects
Source: Biol Open. 2013 Aug 14;2(10):1013–21. doi: 10.1242/bio.20135546 (PMC3798184; doi:10.1242/bio.20135546)
Supplement: Supplementary Material [file supp_2_10_1013__index.html]

Ethanol exposure disrupts extraembryonic microtubule cytoskeleton and embryonic blastomere cell adhesion, producing epiboly and gastrulation defects — Supplementary Material 

# Ethanol exposure disrupts extraembryonic microtubule cytoskeleton and embryonic blastomere cell adhesion, producing epiboly and gastrulation defects

## bio.20135546 Supplementary Material

**Files in this Data Supplement:**

- Supplementary Material - Swapnalee Sarmah et al. doi: 10.1242/bio.20135546
- Movie 1 - **Time lapse movie of control zebrafish embryo from 4.5–5.5 hpf.** Time lapse sequence of embryo is shown with combined DIC and fluorescence images to show injected FITC-labeled histone-1 to identify nuclei. The image sequences are shown 3 times: the first part shows sequences with no markings and no description; In the second part, representative cells in the upper and lower levels are marked to show an interior layer cell (highlighted with dotted line) as it enters the exterior layer (highlighted with solid line); In the third part, brief descriptive points are added to better highlight specific cell movements during intercalation events. LP, lamellipodia; RI, radial intercalation.
- Movie 2 - **Time lapse movie of ethanol treated (from 2–5.5 hpf) zebrafish embryo from 4.5–5.5 hpf.** Time lapse image sequence is shown with combined DIC and fluorescence images to show injected FITC-labeled histone-1 to identify nuclei. The image sequences are shown 3 times: The first part shows sequences with no markings and no description; In the second part, representative cells in the upper and lower levels are marked to show an interior layer cell (highlighted with dotted line) as it enters the exterior layer (highlighted with solid line); In the third, brief descriptive points are added to better highlight specific cell movements during intercalation events. LP, lamellipodia; RI, radial intercalation.
- Table S1 - **Complete list of genes identified at 8 hpf using Affymetrix GeneChip microarray analysis after ethanol exposure (2–8 hpf).**
